# The History of Rabies in Trinidad: Epidemiology and Control Measures

**DOI:** 10.3390/tropicalmed2030027

**Published:** 2017-07-11

**Authors:** Janine F. R. Seetahal, Alexandra Vokaty, Christine V.F. Carrington, Abiodun A. Adesiyun, Ron Mahabir, Avery Q. J. Hinds, Charles E. Rupprecht

**Affiliations:** 1 Department of Preclinical Sciences, Faculty of Medical Sciences, The University of the West Indies, St. Augustine Campus, St. Augustine, Trinidad and Tobago; christine.carrington@sta.uwi.edu; 2 Pan American Health Organization, Trinidad and Tobago Country Office, St. Clair, Trinidad and Tobago; vokatyal@paho.org; 3 School of Veterinary Medicine, Faculty of Medical Sciences, The University of the West Indies, St. Augustine Campus, St. Augustine, Trinidad and Tobago; abiodun.adesiyun@sta.uwi.edu; 4 Department of Geography and Geoinformation Science, George Mason University, Fairfax, VA 22030, USA; rsmahabir@gmail.com; 5 Caribbean Public Health Agency, 16–18 Jamaica Boulevard, Port of Spain, Trinidad and Tobago; hindsave@carpha.org; 6 The Wistar Institute, Philadelphia, PA 19104, USA; charleserupprechtii@gmail.com

**Keywords:** rabies, Trinidad, Caribbean, public health, vampire bat, *Desmodus rotundus*, bat-transmitted rabies, epidemic, zoonosis, One Health

## Abstract

Vampire bat-transmitted rabies was first recognized in Trinidad during a major outbreak reported in 1925. Trinidad is the only Caribbean island with vampire bat-transmitted rabies. We conducted a literature review to describe the changing epidemiology of rabies in Trinidad and give a historical perspective to rabies prevention and control measures on the island. The last human case of rabies occurred in 1937 and although no case of canine-transmitted rabies was reported since 1914, sporadic outbreaks of bat-transmitted rabies still occur in livestock to date. Over the last century, seven notable epidemics were recorded in Trinidad with the loss of over 3000 animals. During the 1950s, several measures were effectively adopted for the prevention and control of the disease which led to a significant reduction in the number of cases. These measures include: vampire bat population control, livestock vaccination, and animal surveillance. However, due to lapses in these measures over the years (e.g., periods of limited vampire control and incomplete herd vaccination), epidemics have occurred. In light of the significant negative impact of rabies on animal production and human health, rabies surveillance in Trinidad should be enhanced and cases evaluated towards the design and implementation of more evidence-based prevention and control programs.

## 1. Introduction

Rabies is a neglected viral zoonosis of major public health and veterinary importance, present in more than 100 countries and territories worldwide [1,2,3,4]. Although vaccine-preventable, this disease is estimated to cause in excess of 60,000 annual human deaths worldwide, with the vast majority in Africa and Asia [5]. Rabies viruses are maintained and transmitted by several mammalian hosts, primarily carnivores and bats [6]. In the Americas, bats are significant reservoirs of rabies viruses, and although rabies virus can affect any species of bat, vampire bats are considered an especially effective vector due to their haematophagous nature [1,7,8]. Vampire bat-transmitted rabies is on the increase in the tropical Americas [9], where it is considered a limiting factor to livestock production [10,11], causing the death of more than 100,000 cattle annually at costs likely exceeding US $30 million dollars [5]. 

Bovine rabies outbreaks (only later attributed to vampire bats) were first reported in the Americas during the late 16th century in Guatemala [12]. Similar incidents were subsequently observed in Ecuador, Brazil, and Trinidad [13,14]. In 1931, the scientific correlation between bats and human rabies was demonstrated in Trinidad, during a historical multi-species rabies outbreak [15,16,17]. Subsequent to this, the disease was diagnosed on the South American mainland with increasing prevalence, and was noted to have affected at least 18 countries by 1968 [1,11,16,18,19,20,21].

The twin island republic of Trinidad and Tobago is located off the northeastern coast of South America, lying approximately 12 km from Venezuela [22]. Trinidad is the only Caribbean island with vampire bat-transmitted rabies. To date, Tobago (which lies 42 km to the northeast of Trinidad [23]), remains free of vampire bats [22], hence, bat-transmitted rabies has not been reported on this island [24]. Given its importance to the natural history of bat-transmitted rabies, the objective of this study was to present a descriptive summary of the epidemiology and control of rabies in Trinidad and to provide a historical perspective to the current measures for prevention and control of the disease on the island. Specifically, we aimed to describe how the overall burden of the disease has changed over the last century in relation to the control and prevention measures implemented and use this to inform future prevention activities for this zoonosis.

## 2. Methodology

A literature review was conducted on rabies in Trinidad from the first reported case of the disease onwards. We leveraged the use of various well-established electronic databases, which included PubMed, WHOLIS (World Health Organization Library Database), SciELO, ScienceDirect, and the AFPMB (Armed Forces Pest Management Board). Key words included “rabies”, “epidemiology”, “rabies epidemic”, “rabies epizootic”, “Trinidad”, “bat rabies”, “bat-transmitted rabies”, “*Desmodus rotundus*”, and “paralytic rabies”. Multiple references also were sourced from the West Indiana Special Collection of the Alma Jordon Library, University of the West Indies, St. Augustine Campus, and the Library Collection at the Caribbean Public Health Agency (formerly the Caribbean Epidemiology Centre), Port of Spain, Trinidad. Supplemental case information, livestock vaccination and human post-exposure prophylaxis data were also collected from personal accounts of field investigations of contemporary animal cases, where possible. 

## 3. Epizootiology and Epidemiology in Susceptible Hosts

### 3.1. Terrestrial Rabies in Trinidad

Historically, rigid quarantine laws were employed in Trinidad whereby all dogs and cats entering the British colony (apart from those from the United Kingdom) were subjected to at least six months quarantine and inspection with subsequent certification by a Government Veterinarian [25]. The last confirmed case of canine-transmitted human rabies (classical hydrophobia) in Trinidad was in 1912. No case of this type of rabies in dogs or other carnivores has been reported since 1914 [15,26,27,28]. In contrast, up to 1993, canine rabies was still a significant problem in the western region of Venezuela, where a minimum of 468 cases of canine-transmitted rabies occurred between 1989 and 1993 [29]. 

Despite the prevalence of rabies in the mongoose population in Grenada and other Caribbean islands (Puerto Rico, Cuba, and Hispaniola), to date, more than a century after the introduction of the small Indian mongoose (*Herpestes auropunctatus*) into Trinidad (1870s), this invasive species still remains on record as rabies-free [30]. In 1955, Dr. Malaga-Alba, a World Health Organization (WHO) rabies expert, detected Negri bodies in a mongoose carcass during his visit to Trinidad [31]. However, island-wide surveys conducted during the same year, and later in 1957 to assess the likelihood of the mongoose as vector for rabies in Trinidad, did not yield any evidence of rabies virus in this species [31,32]. Therefore, it is possible that either the earlier (1955) case may have represented a false positive or the sample size of the second study was not sufficient to detect the virus in the population.

### 3.2. Bat-Transmitted Rabies in Trinidad

The first documented human outbreak of bat-transmitted rabies occurred in Trinidad, during the first half of the 20th century. At the time the first human case was diagnosed, in 1929, the disease was already occurring in the cattle population, but was misdiagnosed [17,27]. A historical multispecies vampire bat-transmitted outbreak followed (1925–1937) that recorded the deaths of approximately 73 humans and thousands of livestock [24,26,33]. Although animals deaths attributed to poisoning were recorded from 1923 in the northwest and southwest of the island, animal deaths ascribed to an infectious agent began during July 1925 with cattle dying on the northwestern Government pastures around the capital of Port of Spain, recording a cumulative herd mortality of approximately 20% [16,17,26]. The disease spread further within the colony for the next four years until 1929, when there was a sudden spike in livestock mortality in the two southern villages of Siparia and Fyzabad [16]. In July of that year, the first documented case of bat-transmitted human rabies occurred in Siparia. The disease advanced within the human population and by the end of 1929 there was a total of 13 human cases [17]. 

**Figure 1:**
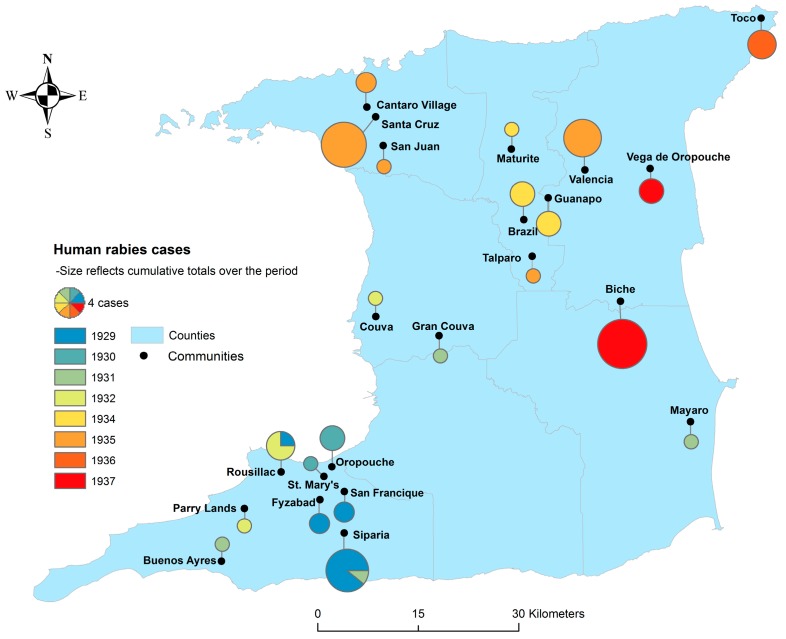
Geographic locations and number of cases by year for bat-transmitted human rabies cases in Trinidad during the period of 1929–1937.

During the period of 1929–1931, when the disease was first recognized, over 1000 animals per year (mainly cattle) were estimated to have died from rabies [15]. However, the actual mortality figures (based on clinical signs) that were recorded were substantially lower [16]. Laboratory confirmation was employed for animal cases from 1931 onwards. Animal cases occurred throughout the island, but were most prevalent in the southern districts until 1933 when a shift occurred in the geographical distribution of cases, from south to north [34]. Human case distribution followed a similar trend until 1935, when disease prevalence peaked with cases occurring principally in the northern community of Santa Cruz [16,17,34], as illustrated in Figure 1. During the human epidemic the highest numbers of cases were reported in Siparia (9), Santa Cruz (10), and Biche (12), and the disease progressed from southwest to northeast within the island [35]. The last human rabies case was in 1937 [24,28,36]. At this time, there was a notable decline in rabies prevalence in the animal population, with approximately 74% fewer cases than the previous year [34]. The clinical disease in humans was typically acute myelitis with spreading (flaccid) paralysis of the lower or upper limbs depending on the bite site [17,33]. Incubation was usually 3–6 weeks with paresthesia (numbness and tingling) and paresis preceding paralysis [17,33]. Other common symptoms included pyrexia (99–104 °C), urine retention, constipation, and profuse salivation and perspiration [17]. Disease duration was generally 6–10 days resulting in death and laboratory confirmation was made by the visualization of Negri bodies in brain tissue [17].

**Figure 2:**
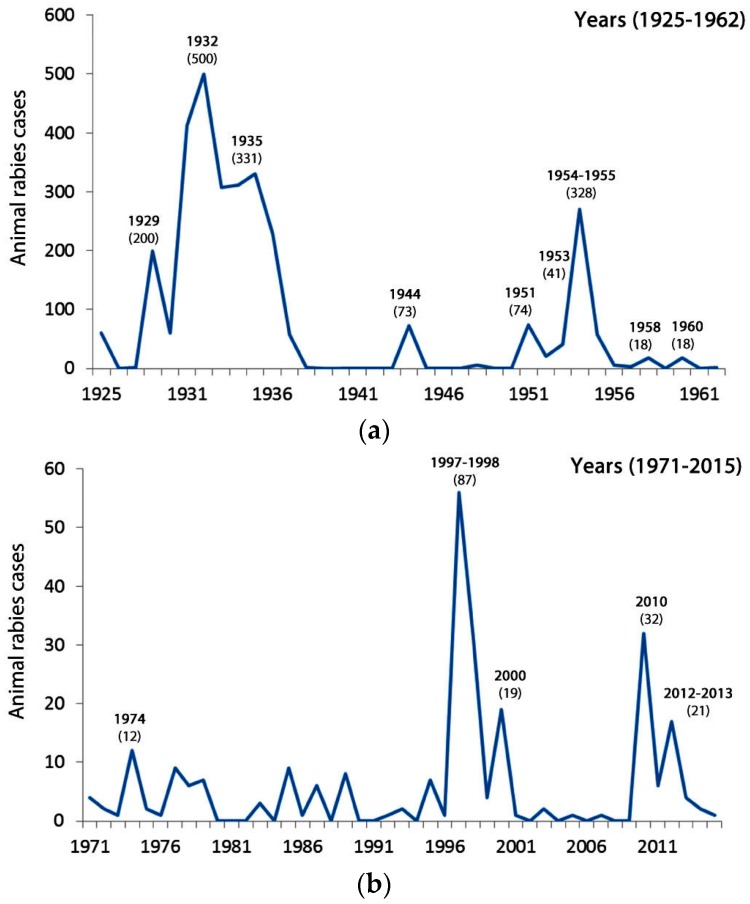
Animal rabies cases reported in Trinidad during the periods (**a**) 1925–1962 and (**b**) 1971–2015.

**Figure 3:**
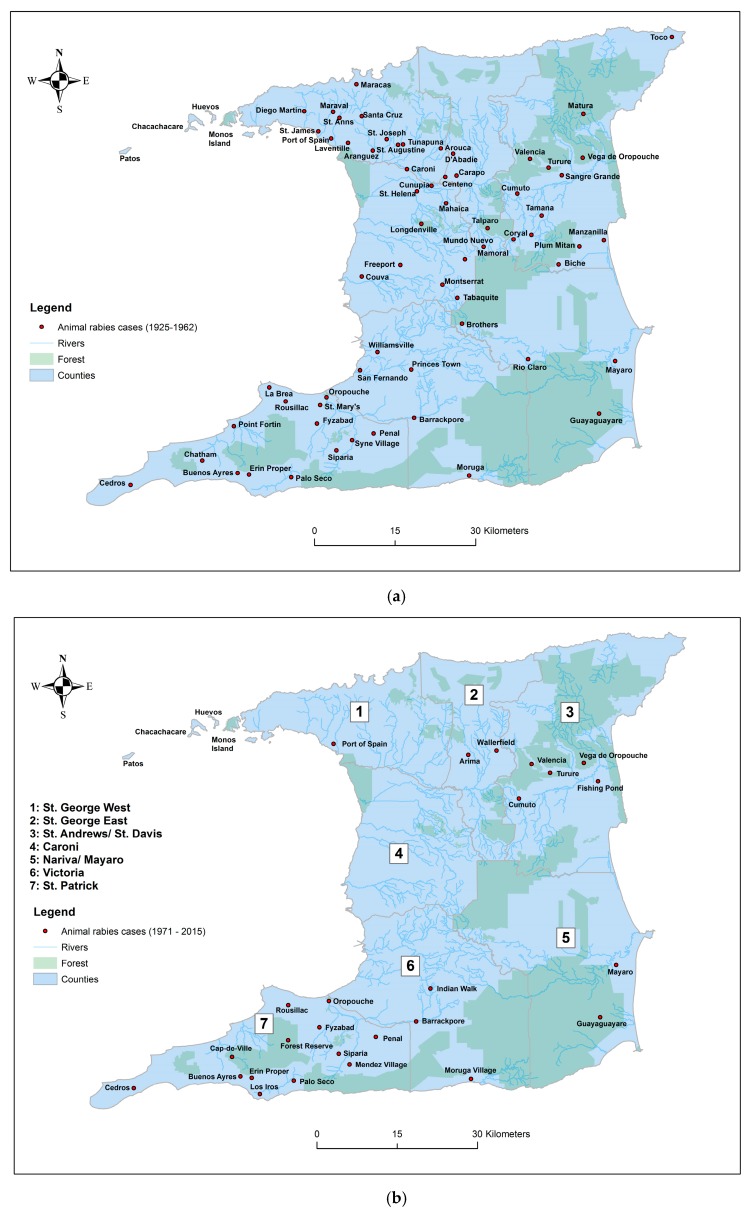
Geographic locations from which animal rabies cases were reported in Trinidad during the periods (**a**) 1925–1962 and (**b**) 1971–2015.

After 1937, a reduced prevalence of rabies was observed in the resident animal population, with sporadic outbreaks every few years [16,24,34]. As illustrated in Figure 2, large outbreaks were noted during 1944, 1951, 1954–1955, 1997–1998, and 2010, with smaller events occurring in 1953, 1958, 1960, 1974, 2000, and 2012–2013 [16,24,34,37]. During 1944, 73 bovine cases were documented in the central and northeastern regions of the island followed by a shift to a southern geographic distribution with a similar sized outbreak in 1951, a smaller outbreak in 1953 (41 cases) and a total of 328 rabies cases (89% bovine) between 1954 and 1955 during a major epizootic event [31,34]. During the smaller 1958 event (18 cases), in addition to cattle, cases also occurred in goats, donkeys, and even pigs [38]. In contrast, a comparable sized outbreak two years later (1960) only affected the bovine population [38]. Figure 3a illustrates the distribution of animal cases from the first observation of the disease until 1962 around which time there was an apparent decrease in the geographic range of cases. Figure 3b illustrates the distribution of animal cases from 1971–2015 which, when compared to Figure 3a, demonstrates the decrease in the geographic range of cases.

After a single case of bovine rabies in 1962 [39], limited information was available on cases until 1971. One study reported two cases of rabies from Trinidad in 1965 [40], and another noted that there was a low sporadic incidence of the disease on the island from 1968 to 1985 [21]. In 1971, four bovine cases were diagnosed [37] by the Veterinary Diagnostic Laboratory (VDL) of the Ministry of Agriculture, which assumed responsibility for rabies diagnostics in 1956 [39]. Initially, diagnostic testing in Trinidad was conducted by histopathological examination of brain tissue for Negri bodies, and intracerebral inoculation of mice with the brain tissue homogenate [15,26,41,42]. However, these methods were phased out and replaced by the direct fluorescent antibody (DFA) test, which was implemented at the VDL in the 1970s [43].

The number of rabies cases decreased proportionally until 1974 when there was an abrupt spike of 12 ruminant cases [37]. Thereafter, cases occurred sporadically, primarily in the southwestern area of the island, until 1997 [37]. An epizootic spike occurred during 1997–1998 consisting of 87 cases (90% bovine), mainly from the northeast region of the country, particularly Wallerfield (76%) [37]. In 1999, the disease again appeared inthe south with fourcases (including one bat) confirmed from Guayaguayare in the southeast [37]. In 2000, 19 bovine cases were diagnosed [37], mostly from the southern villages of Fyzabad and Mayaro. Isolated cases of bovine rabies occurred from 2001 to 2007 in the northeast (Valencia and Fishing Pond) and southwest (Barrackpore and Siparia) regions, until the most recent epizootics in 2010 (32 cases) and 2012–2013 (21 cases), which consisted of ruminant cases and one bat case in 2012, with a notable increase in the prevalence of small ruminant (caprine and ovine) cases, occurring primarily in the southwest particularly from the town of Penal [37]. 

During the 2010 epizootic, 91% (21 cases) of confirmed animal rabies cases for which age was recorded (*n* = 23 cases) were ≥4 months and eligible for rabies vaccination under the national rabies vaccination program [44]. Forty-nine percent (14 cases) of confirmed cases (*n* = 29) for which vaccination status was recorded were eligible for vaccination due to their age but not vaccinated [44]. For the 2012–2013 event, 85% (17 cases) of confirmed cases were eligible for vaccination, but only 15% (three of 20) were documented as being vaccinated [44]. Animal vaccination data was not available for the previous epizootics. The only epizootic event for which human rabies post-exposure prophylaxis (PEP) data was available was in 2010. During this event, 54 humans (82% male) received rabies PEP within the main outbreak area of county St. Patrick, the majority (41 persons) were between the ages of 22–63 years old [45]. Only 72% completed the full course of vaccination, while 28% did not complete PEP [44,45]. Rabies immune globulin was not administered, as it was neither warranted due to the categories of exposure nor available locally at that point in time [44]. No human rabies cases occurred. 

## 4. Rabies Control and Prevention Strategies 

Over the last century, bat-transmitted rabies in Trinidad has evolved from a mysterious syndrome causing significant loss of both human and animal life to a vaccine-controllable disease of ruminants. As a prototypic zoonosis, the prevention and control of rabies is mainly targeted at the animal host. In this light, historically, several strategies have been adopted to prevent rabies in Trinidad. These focus on surveillance, vampire batcontrol, and livestock vaccination strategies, and will be discussed herein within the context of the epidemiology, socio-cultural factors, and natural history of the disease in Trinidad.

### 4.1. Vampire Bat Population Control

Trinidad was the first country to administer a government program for the control of vampire bats. The Bat Control Unit was established during 1934 under the Medical Department, but due to the predominance of livestock cases after 1937 it has since been relocated to the Ministry of Agriculture, where it currently resides as the Anti-Rabies Unit [34,46]. Vampire bats are the primary focus of control efforts as their haematophagous feeding upon mammals favors viral transmission [15]. In Trinidad, two species of vampire bats are indigenous, *Diaemus youngi*, and the more common *Desmodus rotundus* [22]. As previously noted, no vampires have been reported in Tobago [22,24,47]. Methods of vampire bat control have been studied extensively and implemented in Trinidad, based on correlations between the ecology and behavior of these bats and rabies outbreaks [24,26,48,49,50,51]. The control method presently promoted is the topical application of an anticoagulant (most commonly warfarin) paste to the vampire bats, which then return to the roost where they contaminate others in the colony, leading to decimation of the roost population [52].

In Trinidad, culling resulted in an approximately 88% reduction in the annual numbers of *Desmodus* bats caught from 1936 to 1942 [34] The average annual cull rate of 2000 *Desmodus* bats during this period was estimated to have saved 3720 gallons of livestock blood per year and presumably reduced rabies virus transmission [10]. In recent times the annual vampire bat cull rates have been more conservative (e.g., 812 bats caught in 2002 compared to 2136 in 1971) [44]. This could be the cumulative effect of years of successful vampire population control programs with decreased overall population size leading to fewer bats being caught and a reduction in trapping efforts as disease priority and resource allocation dropped. For example, since about 2004, night trapping and anticoagulant pasting of vampire bats have been limited and was further challenged in 2006 by a shortage of anticoagulant paste in Trinidad [44]. This, in turn, appears to have precipitated increased reports of vampire bat-biting in livestock (e.g., from 2007 to 2009 there was an increase of 1369 reported bat biting cases in livestock) [44] cumulating in the 2010 epizootic. 

Reduced cull rates have been manifested by observations of increased numbers of vampires in known roosts and increasing bite rates in both animals and humans. While vampire vector control by culling may pose a challenge to the conservation of biodiversity and some studies have indicated a possible increase in circulating virus due to the elimination of virus immune adults [53], there are currently no known feasible alternatives. Therefore, vector control by culling is the practice currently applied by all countries with vampire bat-transmitted rabies. Although genetic evidence for male-biased dispersal of rabies virus [54] may allow for selective culling, more ecologically sustainable approaches should be actively sought for the long term. It is also recommended that personnel charged with conducting control measures be properly trained in bat identification to avoid decimation of other bat species essential to pollination, seed dispersal, and insect control [55]. Some potential avenues to explore include a topical oral vaccine delivered in a similar fashion to the anticoagulant paste, a vaccine introduced to livestock which inoculates the vampire bats during feeding and prey management strategies to selectively exclude populations from depredation [56]. 

### 4.2. Animal Vaccination

In 1932, mass animal inoculations against rabies were initiated in Trinidad utilizing a locally-produced carbolised brain tissue vaccine, which was eventually phased out and replaced by a commercial purified chicken embryo cell vaccine [34]. In 1956, the Paralytic Rabies Regulations was enacted, which made animal rabies vaccination mandatory and stipulated a penalty charge for contravention [57]. Rabies livestock vaccination is currently provided by the government free of charge and only one commercially-available (inactivated cell culture based) rabies vaccine formulation has been used for this purpose in Trinidad for over 20 years. Due to the feeding habits of the *D. rotundus* vampire bat, bovine rabies cases typically predominate on the island [7,37]. As such, the bovine population has been the traditional target for rabies immunization in Trinidad, although recent increases in case attack rates for the caprine population [37] would suggest value in routinely vaccinating other livestock species. Vaccination was also recently extended to exotic mammals from private zoo collections (e.g., camels, llamas, zebras) as vampire biting was observed on these animals. In light of increased bat biting, further consideration should also be given to include hunting dogs in the national rabies vaccination scheme.

Most of the animal rabies cases that have occurred in Trinidad were not vaccinated for the disease. However, in some instances, as noted in 2010 and 2012, cases were reported in apparently vaccinated animals. These may represent situations of vaccine failure due to improper storage and handling of the biological agents, improper vaccine administration, variability in host immune-competence, or late administration of the vaccine during the course of natural infection. An example of the latter scenario was the single vaccinated case in 2012, in which the vaccine was administered less than one month prior to the development of clinical signs. In this case, the immune response would not have had sufficient time to produce adequate antibodies to combat natural infection considering the peak rabies viral neutralizing antibody response is typically 28 days post vaccination [58]. Vaccine efficacy may also be affected if vaccination regimes differ from the manufacturers’ recommendations due to factors such as the interference of the immune response by maternal antibodies. All these factors must be individually examined to determine the causes of variations in vaccine efficacy and decide upon appropriate action (e.g., education on proper vaccine handling, storage and administration) to rectify gaps in coverage. In 2015 the rabies vaccine coverage for the bovine population was estimated to be 70% [59] but this level is recognized to vary on a year-to-year basis, depending on resources available to deploy the annual rabies vaccination program. Additionally, records document that the vaccine population coverage does not necessarily reflect the level of herd immunity, especially given the possibility of vaccine failure. For example, in 2009, only 2780 rabies vaccines were dispensed for livestock vaccination island-wide [44,60], despite a bovine population estimate of 19,088 animals [61]. This would have represented less than 50% coverage of the bovine population, and may have contributed to the 2010 epizootic event. Current estimates of bovine rabies vaccine coverage from the field are around 40–50% [62], which maybe sub-optimal to afford herd immunity. 

In 2013, a decision was taken to abolish the quarantine of imported dogs and cats. Since then an import protocol for dogs and cats entering Trinidad from canine rabies-endemic countries, including mandatory rabies vaccination and serological testing (to prove a protective rabies titer response), is the main method of canine-rabies prevention by precluding entry of the virus. Import control in Trinidad and Tobago is aided by exclusive sea borders and legislatively supported under the Animal (Importation) Control Regulations under the Animal (Diseases and Importation) Act (1954) [63]. However, these measures do not protect against the introduction of rabies virus by bats.

### 4.3. Surveillance

#### 4.3.1. General Rabies Surveillance Activities and Surveillance in Livestock

Rabies surveillance in Trinidad includes active and passive activities, both of which depend heavily on the effectiveness of reporting systems. Active surveillance is conducted in the *D. rotundus* bat population, while passive surveillance is conducted for all mammals with particular emphasis on livestock. Epidemiological surveillance in livestock entails the reporting of animals being attacked by vampire bats or that are clinically suspected as being rabid. The dependence of rabies surveillance on passive reporting systems may likely result in under-reporting and under-estimation of the actual number of animal cases in general. Other factors which may contribute to the under-reporting of animal rabies cases include: (i) farm inaccessibility to the veterinary services; (ii) limited farmer knowledge of disease etiology and reporting protocols; (iii) cases in small ruminants which are more easily lost or buried; (iv) misdiagnosis of cases (e.g., canine distemper in dogs and tick fever in ruminants); and (v) missed cases due to lack of surveillance in wildlife populations.

#### 4.3.2. Surveillance in the Bat Population

Bat biting case reports allow for the identification of areas with high vampire bat activity and facilitates active bat sampling and the implementation of vampire bat control measures. Areas on the island where high vampire activity is usually noted include Penal and Barrackpore in the southwest, Valencia and Wallerfield in the northeast, and valleys along the foothills of the Northern Range, such as Maracas, Santa Cruz, and Maraval [34]. As a result, these areas have historically demonstrated high densities of rabies cases [34,37]. Earlier uncorroborated studies conducted in Trinidad, suggested that, although rabies causes aberrant behavior and death in bats, apparently healthy vampire bats could also harbor and transmit the virus for extended periods [26,41,42]. On this basis, active surveillance of *D. rotundus* populations was established and is still conducted in parallel with vector control activities. However, virus isolation is rare. For example, during the period 1971–2015, only two of 4399 bats tested were diagnosed rabid by DFA testing [37]. This represents a slightly higher rabies positive rate (0.05%) than previous findings (0.03%), but less than earlier reports of up to 3.3% [41,64,65]. Local testing mainly targets apparently healthy vampire bats in areas of high bat activity (as guided by reports of bat biting cases). On the other hand, early passive surveillance studies conducted on non-vampire bats in the United States found viral isolation rates of 76% in sick bats [66], and approximately 5–6% positivity in all bats tested [66,67]. This may perhaps indicate lower viral circulation in vampire bats due to immunity, although, higher rates would be expected with passive surveillance as mainly sick or moribund bats would be tested. Other than the two vampire species, rabies virus has been isolated from seven other bat species in Trinidad (*Carollia perspicillata*, *Artibeuslituratus*, *Artibeus jamaicensis*, *Molossus molossus*, *Diclidurus albus*, *Pteronotus davyi*, and *Pteronotus parnellii*) [15,24,41,48,68,69] under earlier active surveillance programs. In a more recent study, *Desmodus* variant viruses were found to be the cause of Trinidadian rabies epizootics [64]. Other bat variants have not been documented and, therefore, transmission to other mammalian species from non-vampire bat species has also not been conclusively documented in Trinidad. 

Routine monitoring of vampire bat populations for the presence of rabies viruscould potentially provide an early warning of the risk of virus transmission to a susceptible host. Alternatively, since virus isolation in the bat population is often difficult, rabies antibody titers can be monitored for increases which may indicate increased virus exposure and risk of spill-over to livestock [37]. Epizootics of rabies in vampire populations have been suggested to occur at most every four years with variable viral levels at different stages of the epizootic event [70,71]. However, further studies need to be conducted in the Trinidadian vampire bat population to confirm the frequency of epizootics and the relationship between virus and antibody levels and risk of viral transmission.

#### 4.3.3. Surveillance in the Canine Population

Trinidad remained a British colony until 1962 [72], so the early date of canine rabies elimination (1914), when compared to neighboring Latin America, may have been attributed to the elimination of canine rabies in Britain during 1903 [73], given the implementation of similar disease control measures. Additionally, although rabies has been enzootic on Trinidad since the early 20th century, despite reports of bat biting in dogs (particularly hunting dogs), no cases of rabies have been documented in this population since the 1930s [16]. However, no routine surveillance is focused presently on detecting rabies virus in the dog population of Trinidad. Recent reports of bat biting in dogs [74] underscores the importance of having protocols in place for dealing with a potential canine rabies case. Typically, when not available for testing, wild mammalian reservoirs, such as bats, are regarded as rabid [75] and the animals they bite are considered to be exposed to the virus. As the resident dog population is not routinely vaccinated against rabies, an exposed dog would likely be unvaccinated. Given that there are currently no biologics licensed for post-exposure prophylaxis of unvaccinated domestic animals, and since evidence suggests that vaccine alone is not reliable for prevention of disease in these animals, it has been recommended that unvaccinated exposed dogs should be euthanized [76]. Alternatively, the dog can be held under strict quarantine and observation for four months (with immediate post-exposure rabies vaccination) or six months (if vaccine administration is delayed past 96 h post-exposure) [75]. However, if signs suggestive of rabies develop while under quarantine, the animal should be immediately euthanized and the brain submitted for rabies testing [75]. Stray dog management protocols should also be enforced, particularly in rural forested areas where the risk of bat biting in dogs is higher. A serological approach may also be taken to monitor rabies exposure in this population, which would determine the risk of rabies virus transmission from bats to dogs and can inform preventative measures. 

#### 4.3.4. Surveillance in the Mongoose Population and Other Terrestrial Wildlife Populations

Unlike several other Caribbean islands [30], mongoose rabies has not been documented in Trinidad. Relative to these islands, Trinidad has a lower mongoose population density, which may have allowed the population to remain apparently disease-free [77]. Alternatively, cases could go unrecognized due to the small carcass size and rapid decomposition under the tropical conditions. In Latin America, an increased risk of rabies virus transmission from wildlife has been noted [78,79]. Additionally, in North America, the first case of natural infection of an armadillo with a skunk rabies virus variant and multiple spillover events from bat rabies viruses into foxes and skunks, demonstrates the possibility of rabies virus infection in non-conspecific mammalian taxa [80]. In light of these findings, local studies should be undertaken to determine if there are unrecognized terrestrial (wildlife) reservoirs for sylvatic rabies. The possibility of the initiation of a new virus-host relationship with sustained propagation in a species previously considered a dead-end mammalian host should not be precluded, and monitoring of various potential host species, (e.g., mongoose, ocelot, etc.) is recommended to identify the emergence of new viral reservoir hosts. In this regard, passive surveillance could be used to gather preliminary information which could then inform the development of more structured surveillance activities. As suggested above, serology may also be used in tandem with viral surveillance in the mongoose and other terrestrial wildlife populations to determine the extent of viral exposure and detect possible host shifts from bats. 

### 4.4. Movement Control of Bats and Rabies Viruses 

The geographic spread of rabies virus during epidemic events may be a result of the movement of the animal hosts or vectors. In the case of the former, animal movement control may curtail the spread of the disease. In Trinidad, there are regulations prohibiting the movement of potentially-rabid animals [57], but there is not enough manpower to enforce these regulations on a day-to-day basis so the appearance of livestock cases outside of the main outbreak areas usually represent human-mediated animal movement. On the other hand, viral spread facilitated by bat movement is much more difficult to control than with domestic species, as vampires from different districts visit communal feeding grounds and common livestock feeding may expedite district to district spread of the virus [26]. Nevertheless, spread of the virus during an outbreak event may be limited by the implementation of ring-vaccination of livestock around the index case(s). At present the maximum radius for ring-vaccination activities during outbreaks in Trinidad is eight miles around the index case [62]. Given a reported flight range of about 12 miles for the common vampire bat, the optimum range for ring-vaccination during an outbreak is recommended to be up to 12 miles around the last animal case [7,81]. A southwest–northeast pattern of intra-island virus progression has been observed for rabies epidemics in Trinidad [35,37]. As can be illustrated by Figure 3, over the years the geographic distribution of cases has narrowed to the southwestern regions of St. Patrick and Victoria and northwestern regions of St. Andrew/St. David and St. George East. These are areas with high-density livestock farming at forest fringes where numerous roosting sites may be found in hollow trees [37]. It is also possible that passive surveillance is more effective in these areas because their larger animal populations trigger more efficient case reporting. 

Rabies virus importation to Trinidad from the South American mainland, was proposed by early researchers who also suggested that the virus was first introduced to Trinidad via this route around 1925 [42]. It is plausible that this initial introduction, considering the location of the first animal cases, occurred through the northwest peninsular by bats flying along the island chain (Patos, Chacahacare, Huevos and Monos Islands) between Venezuela and Trinidad (see Figure 3), with the first island only 2.5 miles from the mainland [24]. Subsequently, the northwest–southwest progression of animal rabies cases from 1925–1929 may have represented the initial flight path of infected bats possibly driven by the availability of food sources. Thereafter, human cases followed a southwest to northeast unidirectional pattern, previously hypothesized to be due to bats moving along the Earth’s magnetic field [35]. More recently, a study on the phylogeography of Trinidad rabies viruses provided evidence for at least three independent introductions of virus into Trinidad from the mainland during 1972, 1989, and 2004, and suggested the *D. rotundus* bat as the vector of introduction at the southwestern peninsular of the island with similar northeasterly progression of the virus during epizootics [64]. The flight range for the more common vampire (*D. rotundus*) is 20 km [82], so the distance between Trinidad and Tobago (42 km) [23] (in contrast to only 12 km between Trinidad and the mainland [22]) may preclude the movement of vampire bats between these islands, which would explain the absence of vampire bat rabies in Tobago. Further studies are currently underway to investigate the relationship between rabies virus spatiotemporal dynamics and vampire bat population ecology in Trinidad.

The lack of human rabies cases since 1937, may be attributed to the modernization of housing and associated infrastructure (including indoor and outdoor lighting), which may preclude the free entry of bats into human dwellings. Evidence of this effect may be illustrated by the fact that no rabies cases were reported in urban areas during the human outbreak in the 1930s where housing conditions were generally better than in rural areas, where cases occurred exclusively [35]. Recently, in some rural areas in Trinidad, human bat biting has noted to be increasing, possibly due to an increase in the vampire population. Furthermore, human population expansion into rural areas and the accompanying reduction in total forest cover over the last few decades [83] may result in increased human-bat contact and facilitate viral transmission to vulnerable groups.

### 4.5. Human Vaccinationand Risk Communication Programs

In Trinidad, pre-exposure rabies vaccination is conducted for high-risk personnel (e.g., laboratory staff, veterinarians, and animal health staff), with biennial booster vaccinations. However, ideally, rabies virus neutralizing antibody levels should be monitored every six months or two years in these personnel depending on their risk of exposure, with booster doses if serum titer levels fall below 0.5 IU/mL [5]. The WHO-recommended Essen (five-dose) regimen is used for rabies post-exposure prophylaxis (PEP) of previously-unvaccinated individuals, with the rationale for PEP administration ideally based on the WHO guidelines [2,4]. Using these guidelines, the risk of rabies virus transmission by the handling of suspect ruminant cases is thought to be low, which is further supported by infrequent virus isolation from bovine saliva with bat-transmitted disease [84]. However, during 2006, an unvaccinated Brazilian veterinarian was infected via non-bite exposure while treating a rabid herbivore and died, highlighting the fact that the benefits of vaccination far outweigh the risks [85].

In Trinidad during 2010, the demographic of the cohort of humans administered PEP was consistent with that of small scale livestock farmers and butchers. The level of contact reported for farmers was primarily the handling and attempted treatment of suspect animals, which mainly involved manual (bare-handed) manipulation of the oral cavity of cattle during attempts to remove an assumed foreign body during suspected choking events. Even though the physical handling of a rabid animal on its own is not generally considered an exposure, the potential opportunity for scratches and abrasions from bovine teeth and oral cavity papillae complicated the risk assessment. The risk for butchers was based on possible splash exposure during the slaughtering of potentially-rabid animals. In most instances PEP was administered as a precaution due to the opportunity for exposure. In total, during the 2010 epizootic, 342 doses of human diploid cell vaccine (HDCV) were administered [44,45], which was projected to cost more than US$75,500 using previously published HDCV cost estimates [86]. This figure would also have been higher if rabies immune globulin was needed and if all individuals adhered to recommendations for completion of the full-course of PEP. This emphasizes the relevance of risk communication to occupationally high-risk groups in an effort to reduce such exposures. Apart from the public health risks during animal production and slaughter, other risks are associated with consumption of raw or improperly-cooked meat. In fact, in New Mexico, 4% of cattle slaughtered for human consumption were reported to be infected with rabies, and although no human cases were documented to arise from the meat consumption, dogs which consumed the uncooked meat were noted to develop a paralytic disease [87]. In Trinidad, the carcasses of suspect rabid animals are buried on site at the farm after samples have been taken for laboratory testing and in some instances (especially in cases of sudden death) the whole carcass is buried by the farmer without sampling. Dogs have been known to try digging up these carcasses, especially in cases of shallow burial. This poses a risk to dogs, as well as humans in contact with these animals. 

Rabies educational programs, which are predominately reactive outbreak communications, can incite and scare the recipient population, with detrimental outcomes on the livestock market. Therefore, in Trinidad, where the disease is endemic, a recommended approach to risk communications would be routine rabies awareness programs with precaution advocacy in high-risk areas targeting high-risk groups. This may additionally increase compliance to vaccination programs and reduce the rabies prevalence in the animal population. Public education and awareness activities must also take into account the local cultural practices and provide information in a strategically-targeted, timely manner. For example, large-scale domestic slaughtering of livestock often conducted prior to specific cultural events in Trinidad provides an ideal opportunity for educating butchers on the potential risk of slaughtering rabid animals. Furthermore, the annual observation of World Rabies Day on 28 September presents a unique opportunity to conduct large-scale rabies awareness activities on a predictable basis. 

## 5. Conclusions

Rabies poses major public health and veterinary health challenges in Trinidad. Given the epidemiology of rabies in Trinidad and existing prevention and control measures, rabies epizootics are likely due to gaps in preventative programs, such as prolonged periods of limited vampire bat population control, pockets of unvaccinated susceptible animals, and inadequate public awareness of the disease and the existing preventative measures. Therefore, actions taken to address these shortcomings should include sustained vampire control and vaccination activities (to afford herd immunity) particularly within high risk areas, as well as the implementation of routine public awareness programs. The age of primary vaccination, duration of immunity of vaccines, and the effective range of ring-vaccination efforts during outbreaks need to be evaluated in light of the available scientific data. These actions would not only result in the minimization of livestock losses, but also a substantive reduction in human healthcare costs. Furthermore, surveillance in Trinidad should be enhanced and the results examined actively to elucidate disease trends and risk factors. 

Given the inter-related variable of the environmental, pathogen, public health, and veterinary concerns, future considerations should focus upon enhanced laboratory-based surveillance, epidemiologically sound livestock vaccination and risk-based prophylaxis of exposed humans, as well as novel methods for vampire bat management. In a One Health context, such information would aid in the reduction of rabies risks in both human and animal populations by facilitating the development of efficient evidence-based ecologically-sustainable approaches to disease prevention and control.
